# 

**Published:** 1890-02

**Authors:** 


CONTENTS

Vol. 37 No. 2.

| | PAGE. |
|---|---|
| National Sisterhood of Liberal Women | 25 |
| Looking Forward | 26 |
| Looking Forward More Truly | 31 |
| Rev. M. J. Savage on Occultism | 35 |
| National Liberal Women's Suffrage Association | 38 |
| Various Uses for Ammonia | 38 |
| A Dangerous Drug | 39 |
| Woman | 40 |
| A Mystery | 41 |
| A Social Reform from the Kitchen | 42 |
| How and When to Drink Water | 42 |
| Removal of Moles by Electricity | 43 |
| Laughter as a Health Promoter | 43 |
| A Good Test for Coffee | 43 |
| "Remedies" | 44 |
| Infant Damnation | 44 |
| The Source of Wrinkles | 45 |
| Diseases from a Book | 45 |
| Household | 46 |
| Miscellaneous | 47 |
| Literary | 48 |

INDEX TO ADVERTISEMENTS OF HALF A PAGE AND OVER.

| | PAGE. |
|---|---|
| An Unprecedented Offer | I |
| Celluloid | II |
| Hollow Suppositories | III |
| Christ Before Pilate | IV |
| Lactopeptine | V |
| E. C. Morris & Co | VI |
| Dennin's Certain Cure | VII |
| The Consumers' Supply Association | VIII |
| The Herb Cure Co | X |
| Revised Premium List | XI |

